# Preclinical and Clinical Pharmacology of Hydrocodone for Chronic Pain: A Mini Review

**DOI:** 10.3389/fphar.2018.01122

**Published:** 2018-10-01

**Authors:** Luigi Cardia, Gioacchino Calapai, Domenico Quattrone, Cristina Mondello, Vincenzo Arcoraci, Fabrizio Calapai, Carmen Mannucci, Epifanio Mondello

**Affiliations:** ^1^Anesthesia, Intensive Care and Pain Therapy, Azienda Ospedaliera Universitaria Policlinico “G. Martino” - Messina, Messina, Italy; ^2^Department of Biomedical and Dental Sciences and Morphofunctional Imaging, Azienda Ospedaliera Universitaria Policlinico “G. Martino” - Messina, Messina, Italy; ^3^Pain Therapy Unit, Grande Ospedale Metropolitano Bianchi Melacrino Morelli–Reggio Calabria, Reggio Calabria, Italy; ^4^Department of Clinical and Experimental Medicine, Azienda Ospedaliera Universitaria Policlinico “G. Martino” - Messina, Messina, Italy; ^5^Pharma.Ca Research Facility (Centro Studi Pharma.Ca), Messina, Italy

**Keywords:** hydrocodone, chronic pain, opioids, pain, analgesics

## Abstract

Hydrocodone is one of the most prescribed oral analgesic drugs and it is one of the most abused drugs in general population. It is a mu-opioid agonist predominantly metabolized to the O-demethylated product hydromorphone and to the N-demethylated product norhydrocodone. The purpose of the study is to summarize the preclinical and clinical characteristics of hydrocodone. Pharmacokinetic aspect (terminal half-life, maximum serum concentration, and time to maximum serum concentration) of hydrocodone and the influence of metabolic genetic polymorphism in analgesic response to hydrocodone are also illustrated and commented. Literature on experimental preclinical pharmacology investigating analgesic activity in laboratory animals is furtherly discussed. Moreover, the authors discuss and comment on the updated data regarding safety profile and effectiveness of hydrocodone in the treatment of chronic pain. A bibliographic research was carried out (from February 01, 2018 to August 28, 2018) independently by two researchers (blinded to the authors and initially on results) in the major scientific databases and research engines of peer-reviewed literature on life sciences and biomedical topics, starting from January 1990 to August 2018. Analysis of results of clinical studies suggests that abuse-deterrent extended-release (ER) hydrocodone formulations can be effective and they are well tolerated in the treatment of chronic low back pain. Weaker is the evidence of the analgesic effectiveness of ER hydrocodone on other chronic pain syndromes and non-cancer non-neuropathic chronic pain. In these conditions, hydrocodone showed to have positive effects in non-controlled open studies and needs to be further studied to assess the real strength of results.

## Introduction

Opioids are the most potent drugs producing analgesia and their use is fundamental for the clinical pain management (Mercadante et al., 2014). The largest part of prescriptions regarding pain relievers opioid drugs are oxycodone, hydrocodone, morphine, codeine, methadone and transdermal morphine and fentanyl (Graziani and Nisticò, 2016). Hydrocodone is currently used in the pain management, but risks related to its abuse and misuse raise an increasing problem for health (Kenan et al., 2012; Smith et al., 2013). Objective of this review is to summarize the principal preclinical and clinical characteristics of the opioid drug hydrocodone.

A bibliographic research was carried out (from February 01, 2018 to August 28, 2018) independently by two researchers (blinded to the authors and initially on results) in the major scientific databases and search engines of peer-reviewed literature on life sciences and biomedical topics (PubMed, Scopus, Embase, Web of Science, and Google Scholar) starting from January 1990 to August 2018. The investigators used the following keywords or combination of keywords: “hydrocodone,” “hydrocodone” and “chronic pain,” “hydrocodone” and “opioids.” The analysis included all articles written in English language, published in peer-reviewed scientific journals, describing preclinical findings and clinical applications of hydrocodone. All the authors reviewed all the eligible articles and resolved by discussion any uncertainty regarding the content about hydrocodone to be discussed.

## Hydrocodone in the Medicinal Products

Hydrocodone is a semi-synthetic phenanthrene opiate derivative with analgesic and antitussive effects. The chemical name of hydrocodone is (4R,4aR,7aR,12bS)-9-methoxy-3-methyl-1,2,4,4a,5,6,7a,13-octahydro-4,12-methanobenzofuro[3,2-e]isoquinoline-7-one, the drug name dihydrocodeinone was given when it was first marketed in Germany (Fraser and Isbell, 1950). Since the release, in 1943, of the first product, hydrocodone acquired growing popularity as a drug considered as a “middle-level” opioid (Covvey, 2015). The rescheduling of hydrocodone combination products has been discussed in the United States by Food and Drug Administration (FDA) in 2012. Currently, hydrocodone is listed in Schedule II of the Controlled Substances Act. Following a re-evaluation of the drug abuse-related data, hydrocodone combination products including analgesic and cough suppressant compounds were listed in Schedule III (Food and Drug Administration [FDA], 2012).

It was originally marketed as a single drug as immediate-release (IR) dosage forms indicated for the short management of acute pain and it was successively released in association with non-opioid drugs (Gould and Paul, 2015). Opioids and co-analgesics such as non-steroidal anti-inflammatory drugs are often used to improve pain control or to reduce opioids prescription or their dosage and decrease the risk for adverse events caused by opioids (Vardy and Agar, 2014). In particular, hydrocodone has been marketed in combination with different dosages of acetaminophen to increase analgesia and simultaneously to induce reduction of the intake of hydrocodone because of the acetaminophen side effects. It is well known that excessive doses of acetaminophen are the leading cause of acute liver failure in the developed world (Larson et al., 2005). This combination has been authorized in the United States with different amounts of acetaminophen (200, 325, 400, 500, 650, or 750 mg) with a presence of hydrocodone in the tablets of 2.5, 5, 7.5, or 10 mg, with a dosing interval from 4 to 6 h (Krashin et al., 2013; Gould and Paul, 2015).

Analgesic products containing hydrocodone in combination with the anti-inflammatory drug ibuprofen (hydrocodone bitartrate/ibuprofen 2.5–7.5 mg/200 mg) to be taken by oral administration and a long-acting formulation of hydrocodone not containing acetaminophen were also released (Krashin et al., 2013).

In February 2018, the FDA approved the association between the prodrug benzhydrocodone and acetaminophen (Mustafa et al., 2018). However, therapeutic indication for both these drugs is for the short-term management of acute pain.

Changing hydrocodone from schedule III to schedule II has been associated with an increase in the total amount of opioids filled in the initial prescription following surgery (Habbouche et al., 2018). However, hydrocodone prescriptions decreased, and prescriptions of oxycodone, another opioid drug widely used to alleviate moderate and severe acute and chronic pain, increased in frequency (Pantano et al., 2017; Tan et al., 2018). The necessity to reduce chronic pain produced an increase in use of opioids leading in turn to a growing opioid misuse, abuse and addiction associated with overdose deaths (Volkow and McLellan, 2016). It has been reported that only in United States of America in the year 2012 about 2 million persons were taking for the first time prescribed opioids. The same analysis reported that the most of misused prescription drugs in young people were pain relievers (Graziani and Nisticò, 2016).

Other novel synthetic opioids, such as U-47700, U-49900, AH-7921, or MT-45 have no recognized therapeutic use but emerged as non-illegal drugs diffused to circumvent prohibition laws, resulting in numerous abuse reports and overdose cases, especially across the United States and Europe (Solimini et al., 2018).

Abuse of hydrocodone seems to be lower than the oxycodone abuse. This is true for previously dependent opioid abusers, for non-dependent opioid abusers (Zacny and Gutierrez, 2009) and for non-physical dependent prescription opioid abusers (Walsh et al., 2008).

Hydrocodone is the second drug of abuse in the United States but the first preferred by women. Female preference could be due to the fake perception of a better safety profile deriving by the minor rate of overdose associated with hydrocodone (Cicero et al., 2013). This misconception has been successively denied by subsequent reports showing the increase of hydrocodone-related deaths and by the prevalence of female among the victims (Mowry et al., 2016; Gummin et al., 2017). The grown consumption raised concerning safety issues because, in 2011, use of hydrocodone was linked with about 97,000 drug-related emergency room visits caused by abuse/misuse. With the aim to attenuate this phenomenon regulatory acts were developed about hydrocodone products. After consultation of stakeholders and companies the agency FDA took note that products with hydrocodone or similar opioid products contained larger quantities of drugs in a single tablet to be taken over a short period (8–24 h). In this way, these products showed attractiveness and had a strong potential for abuse and adverse reactions. On this basis, the FDA introduced a risk evaluation and mitigation strategy for extended-release (ER) long-acting opioid products (Covvey, 2015). Moreover, the FDA authorized for long-term management of pain two new oral ER products based only on hydrocodone bitartrate (10–90 mg) (Covvey, 2015). Abuse-deterrent formulations have been designed and marketed with the aim to obtain opioid-based products keeping effective analgesia but the decreasing behavior of abuse through the use of alternative routes of administration (Hale et al., 2016a,b).

ER opioid formulations are considered more suitable for appropriate management of pain in chronic patients. Oral intake of ER medicinal products produces higher plasmatic concentrations and lower peak-to-trough changes over the dosing interval, in comparison with IR products (Gudin, 2013; Nicholson, 2013). The first formulation having, according to FDA, abuse-deterrent characteristics contained only hydrocodone bitartrate was a once-daily hydrocodone bitartrate ER product, prescribed for the long-term opioid treatment of severe pain refractory to other analgesic strategies (Dhillon, 2016).

## Preclinical Studies

Metabolism of hydrocodone was studied in laboratory, animals and species differences have been observed. In rats, metabolic conversion of hydrocodone to hydromorphone is performed by the enzyme CYP2D1, homolog of the human CYP2D6. While hydrocodone mainly underwent O-demethylation and ketone reduction in rats to form hydromorphone and hydromorphone in the reduced form, in dogs it is metabolized prevalently by N-demethylation and N-oxidation (Li et al., 2013).

Rewarding and euphoric effects of hydrocodone, investigated by using the conditioned place preference (CPP) paradigm, have been compared in rats to those of morphine. In laboratory animals, hydrocodone is self-administered and produces an opiate-like subjective discriminative generalization profile and a withdrawal syndrome after sudden treatment cessation that was similar to morphine and/or oxycodone (Gauvin et al., 2015).

Hydrocodone and morphine injected intraperitoneally produce a CPP at the 5 mg/kg dose, but not the lower 1 mg/kg dose, suggesting similar rewarding properties, furthermore hydrocodone and morphine equally reduced phosphorylation levels of extracellular signal-regulated kinase (ERK) and cAMP response element-binding (CREB) proteins in the nucleus accumbens, thus indicating that these drugs cause their effects through signal transduction pathways involved in rewarding and reinforcing effects (Tenayuca and Nazarian, 2012).

Studies *in vivo* in rats by using the pain models “tail withdrawal test” and “formalin test” on opioids administered subcutaneously, shown that analgesia caused by hydrocodone is greater than codeine and lower than, in decreasing order, fentanyl, buprenorphine, oxycodone, and morphine (Meert and Vermeirsch, 2005). Sex differences in antinociceptive effects of opioids have been observed in rats. Male rats are more sensitive to the antinociceptive effects of morphine than female rats and this difference, as a lesser extent, is evident also with hydrocodone (Peckham and Traynor, 2006).

## Pharmacokinetics of Hydrocodone

After single oral ingestion, hydrocodone reaches maximum serum concentrations within 1 h and it shows to have an elimination half-life of 4–6 h (Otton et al., 1993). Following single oral doses of ER hydrocodone formulations, blood concentration reaches the peak (Cmax) at a median time (Tmax) of 14–16 h for the different doses (the range is 6–30 h). Hydrocodone steady state is reached in 2 days after taking once daily an ER hydrocodone formulation (Kapil et al., 2016). Hydrocodone and its metabolite norhydrocodone appear in the urine within 2 h after single drug administration (Cone et al., 2013).

The size of plasma protein-binding is unknown but it could be similar to semi-synthetic opiates such as hydromorphone, about 19% bound (Cone et al., 2015). The apparent volume of distribution after ER administration is 402 L (for an adult of 70 kg), thus indicating a large hydrocodone tissue distribution (Dhillon, 2016).

**FIGURE 1:**
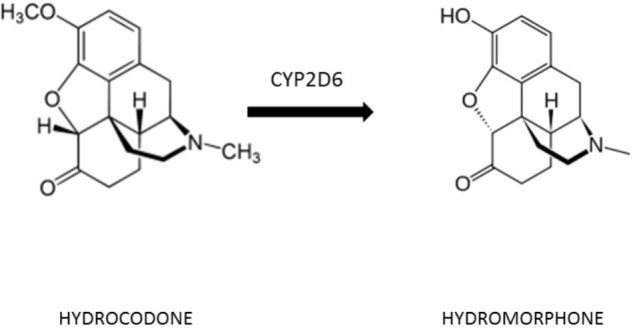
Metabolic conversion of hydrocodone to hydromorphone.

The principal metabolites of hydrocodone are norhydrocodone and hydromorphone. Hydrocodone is a prodrug (inactive), only through the bioconversion to its active metabolite hydromorphone it induces analgesia (Otton et al., 1993; **Figure 1**). Pain relief correlates with plasma hydromorphone but not with hydrocodone concentration, thus confirming that the ability to convert hydrocodone to its active drug is essential (Stauble et al., 2014). Hydrocodone is transformed to hydromorphone through O-demethylation catalyzed by the cytochrome P450 (CYP450) enzyme CYP2D6, influencing the metabolism of 25% of all drug therapies (Cone et al., 1978).

Hydromorphone undergoes phase II glucuronidation to be transformed in the predominate metabolite hydromorphone-3-glucuronide. Approximately 7% of the Caucasian population are poor metabolizers (PMs), causing a slower rate of conversion of hydrocodone to hydromorphone. Urinary hydromorphone after a single dose of hydrocodone was found at relatively small amounts in both extensive metabolizers (EMs) and PMs but PMs were equally responsive to oral hydrocodone as EMs. The study demonstrated that although hydrocodone is less potent than hydromorphone, it clearly has its own agonist actions (Valtier and Bebarta, 2012).

Hydrocodone is also metabolized by CYP3A4, but the product of transformation is the inactive metabolite norhydrocodone (Hutchinson et al., 2004). Isoenzymes CYP2B6 and CYP2C19 may also be partially involved in the formation of norhydrocodone and hydromorphone (Dhillon, 2016).

Approximately 3% of Blacks, 1% of Asians are PMs of CYP2D6. The remainder of individuals in these populations produce functional levels of CYP2D6 and are labeled EMs (Bertilsson, 1995).

Patients who are ultra-rapid metabolizers may produce more hydromorphone, while subjects who are PMs of CYP2D6 may experience little to no analgesia from hydrocodone since they lack or have not sufficient activity of the enzyme to metabolize it (Lurcott, 1998; De Leon et al., 2003). PMs are able to produce only small amounts of the active metabolite hydromorphone, regardless of dose. After a 10 mg oral dose of hydrocodone, hydromorphone levels in EMs have been found to be about 5–10 times greater than in PMs (Otton et al., 1993). Besides CYP2D6 PMs, also patients in therapy with strong inhibitors or high-affinity substrates of CYP2D6 such as the antiarrhythmic quinidine and the antidepressants selective serotonin reuptake inhibitors, paroxetine and fluoxetine, are unable to metabolize hydrocodone into its active metabolites. Even if, prescription in patients of hydrocodone with antidepressant drugs has not been met with any poor analgesic issues in clinical practice, in these patients it can be recommended to prescribe hydrocodone in its active form, such as hydromorphone (Lurcott, 1998). The isoenzymes of P450 system CYP2B6 and CYP2C19 may also be partially responsible for the transformation in the metabolites norhydrocodone and hydromorphone (Pathan and Williams, 2012).

Other products of the hydrocodone metabolism are the glucuronate conjugated products to hydrocodone-3β-glucuronide and hydrocodone-6β-glucuronide. The metabolite hydromorphone is also glucuronidated to hydromorphone-3β-glucuronide and hydromorphone-6β-glucuronide (Larson et al., 2005). The 6β-metabolites but not 3β-metabolites are active and 6β-metabolites are more active than hydrocodone against pain (Fordyce, 1989).

Renal excretion is the principal way of elimination for hydrocodone and its metabolism products. Renal hydrocodone elimination in healthy subjects account for 6.5% and it reduces in values with the severity of the disease (Dhillon, 2016). Severe or moderate liver insufficiency, moderate to severe renal impairment or end-stage renal disease may cause higher plasmatic hydrocodone level. Age and gender seem to be not affecting the pharmacokinetics of hydrocodone (Dhillon, 2016).

Blood concentrations of hydrocodone and its metabolite dihydrocodeine are widely used to determine the cause of death. While concentrations in postmortem samples may not necessarily reflect the original drug concentration at the time of death, a study of liver to peripheral blood hydrocodone ratio suggests that hydrocodone is unlikely to undergo substantial postmortem distribution changes (Saitman et al., 2015).

## Pharmacodynamics of Hydrocodone

Hydrocodone produces its analgesic effects by activating mu-opioid receptors (MORs), it is a μ-opioid receptor agonist analgesic, even though when higher concentrations are reached it can bind with different opioid receptors. As the dose of opioids increases beyond typical starting doses, delta-opioid receptors and kappa-opioid receptors are activated. MORs are G-protein coupled receptors that inhibit cAMP production and activate G-protein mediated inwardly rectifying potassium channels. The analgesic effect appears to be associated with the latter signaling pathway. As the dose of hydrocodone increases over the starting doses, delta-opioid receptors and kappa-opioid receptors are also activated. In *in vitro* experiments, hydrocodone itself is a low efficacy agonist (Mitrovic et al., 2003; Gould and Paul, 2015).

The affinity measured using a single binding assay in a cell membrane preparation expressing recombinant human MOR of the product of conversion hydromorphone for the MOR has been reported to be over 100 times greater than that of hydrocodone, with Ki values of 0.36 nM for hydromorphone versus 41.58 nM for hydrocodone (Volpe et al., 2011; Vuilleumier et al., 2012).

Hydromorphone, but not hydrocodone, exerts analgesic effects (Boswell et al., 2013) and it possesses more potency (7–10 times) than morphine (Stauble et al., 2014).) Approximately 0.9–1.2 mg of hydromorphone is equianalgesic with 10 mg of morphine, with a similar incidence of side effects (Mahler and Forrest, 1975).

Hydromorphone had a similar effect on patient-perceived cancer pain intensity as described for oxycodone and morphine (Bao et al., 2016) and there is not sufficient evidence to support or refute the suggestion that hydromorphone is effective in neuropathic pain (Stannard et al., 2016). As it is for oxycodone and dihydrocodeine, the potency of hydrocodone is about 10 times than its parent molecule, codeine (Gould and Paul, 2015). It is less polar than codeine and thus has more rapid pharmacokinetic properties. Rapid pharmacokinetics influences reinforcing effects and potential of abuse of hydrocodone (Ko et al., 2002; Flugsrud-Breckenridge et al., 2007).

It has also been shown that hydrocodone, as well as morphine, is conditioning the locomotor response involved in the dopamine reward system to the D2/D3 dopamine receptor agonist quinpirole (Emery et al., 2015).

## Hydrocodone for Chronic Pain: Clinical Studies

Opioids are used for moderate to severe chronic non-cancer pain in patients that are refractory to other analgesics such as acetaminophen and nonsteroidal anti-inflammatory drugs and when other opioids are not appropriate in patients because they experience unsupportable adverse effects (Chou et al., 2009).

In 2008, the American Society of Interventional Pain Physicians (ASIPP) released guidelines to provide guidance for opioids use for chronic non-cancer pain. According to these guidelines, hydrocodone and methadone were considered at level III of evidence, the level of evidence for transdermal fentanyl and sustained-release morphine was II-2, whereas for oxycodone the level of evidence was II-3. The level III of clinical evidence for hydrocodone is a weak level since was based on expert opinion (Trescot et al., 2008).

More recently, several studies have been performed to investigate on the effectiveness of ER hydrocodone in chronic pain. Chronic pain has been recognized as pain that persists past normal healing time and hence lacks the acute warning function of physiological nociception (Treede et al., 2015).

The authors collected 13 clinical studies on the effects of ER hydrocodone bitartrate administered alone in the treatment of chronic pain deriving from different pathologic conditions.

Five clinical studies were carried out with ER hydrocodone on chronic pain deriving from low back pain. Four of them were randomized controlled double-blinded clinical trials (RCTs), one was designed as a 22-week open study. The RCTs recruited 1246 patients; dose ranging was 15–120 mg of ER hydrocodone every 12 h, duration of treatment was 12 weeks in all the clinical trials. Similarly, conclusions were that ER hydrocodone is significantly more effective than placebo in alleviating chronic low back pain and shows a safety profile without the risk of liver toxicity associated with acetaminophen (Rauck et al., 2014; Hale et al., 2015a,b; Wen et al., 2015). The open study recruited 182 patients with chronic low back pain receiving ≥1 dose of abuse-deterrent ER hydrocodone (15–90 mg every 12 h), 170 entered open-label treatment for 22 weeks and 136 completed the study. ER hydrocodone was generally well tolerated and maintaining efficacy over the period of treatment (Hale et al., 2016a).

Three studies reported the effects of ER hydrocodone on non-cancer non-neuropathic moderate-to-severe chronic pain. They were long-term open-label designed studies recruiting a total number of 542 patients. Ranging dose of ER hydrocodone was 20–120 mg administered once daily for an elapsed period of 12–18 months. Adverse events reported were those normally associated to opioids: nausea, vomiting, constipation, dry mouth, hematemesis, abdominal pain, dizziness, dysgeusia, headache, myalgia, paresthesias, scratch, fatigue, sleep disorders, and hyperhidrosis (Kapil et al., 2016; Taber et al., 2016; Broglio et al., 2017).

Another open-label, long-term trial (1-year maintenance treatment) investigated on long-term safety and effectiveness of hydrocodone 20–120 mg tablets taken by 269 patients with moderate to severe chronic, non-malignant and non-neuropathic pain. Supplemental non-opioid pain medications were permitted. A total of 226 patients (84%) completed the 1-year maintenance period. Results showed a reduction in pain intensity consistently maintained during the entire treatment period (Lynch et al., 2014).

One study evaluated safety and effectiveness of a once-daily, single-entity, ER hydrocodone over a 52-week period in 97 patients with chronic non-cancer and non-neuropathic pain who required opioid rotation from IR oxycodone. Hydrocodone was well tolerated and produced effective analgesia; furthermore, use of opioids decreased (Pergolizzi et al., 2017).

Other three open studies investigated on the effectiveness of hydrocodone on chronic pain deriving from any origin. Dose ranging was 15–300 mg divided into two daily doses for a period of 3–13 months. Results showed moderate to substantial levels of pain relief associated with functional improvements in patients treated with ER hydrocodone (Argoff et al., 2015; Hale et al., 2015a,b,c).

Finally, Bartoli et al. (2015) showed that hydrocodone was effective in reducing pain intensity and in maintaining analgesia over time without the need for continued dose increase and with tolerability profiles similar to those of other opioid analgesics. Treatment showed positive effects also on the health-related quality of life (HRQL), although not in mental HRQL or sleep quality.

Data from this overview of clinical studies with ER hydrocodone suggest that this formulation can be used to relieve chronic pain. However, more relevant results were showed in RCTs conducted in patients with low back pain. On a lower step of importance are open studies on chronic non-cancer and non-neuropathic pain. All the studies showed a good tolerability of ER hydrocodone and the one long-term open study (Lynch et al., 2014) indicated that both effectiveness and tolerability could be maintained over time.

## Safety Profile of Hydrocodone

Hydrocodone use can trigger the occurring of adverse reactions. Abuse, addiction, and adverse effects related to opioid drugs have been detected (Larochelle et al., 2015). At higher doses, hydrocodone can cause respiratory depression due to direct action on the brain stem centers. As well as occurs with other drugs acting on the central nervous system, hydrocodone may impair mental and/or physical abilities, such as driving a vehicle or operating machinery. The risk for respiratory depression and coma is more frequent at the beginning of therapy with or when the dose of the drug is increased (Cone et al., 2013).

There have been cases of self-reported severe to profound sensorineural hearing loss in people using hydrocodone-acetaminophen association. Sensorineural hearing loss is an ototoxic condition resulting in permanent, severe to profound auditory damage and it has been associated with the use of these combination products (Rigby and Parnes, 2008). Hearing loss does not resolve with withdrawn of hydrocodone or with the application of a steroidal therapy (Ho et al., 2007).

It has been suggested that opioids such as transdermal fentanyl, methadone, and oxycodone can be associated with increased odds of androgen deficiency. However, this kind of risk is lesser with hydrocodone use (Rubinstein and Carpenter, 2017).

## Conclusion

Hydrocodone is one of the most prescribed and effective opioid analgesic drugs, however, the rate of its abuse raised a new health problem. In particular, medicinal products based on association hydrocodone-acetaminophen released with the aim to enhance analgesic effects and at the same time to reduce the dose of hydrocodone caused abuse and addiction prevalently in young people. To fight this phenomenon, abuse-deterrent formulations ER long-acting opioid products were authorized, however, these deterrent forms remain abused orally. Analysis of results of clinical studies considered for this review suggests that abuse-deterrent ER hydrocodone formulations can be effective and they are well tolerated in the treatment of chronic low back pain. However, guidelines for the management of chronic pain still report for hydrocodone the level III of clinical evidence, corresponding only to expert opinion. Although it is important to keep in mind that several studies are from corporate sponsored articles, on the basis of the assessed effectiveness of RCTs investigating on long-term therapy with ER hydrocodone in patients affected by low back chronic pain. About the evidence of the analgesic effectiveness of ER hydrocodone on other chronic pain syndromes and non-cancer non-neuropathic chronic pain, hydrocodone showed to have positive effects in open non-controlled studies and needs to be further studied to assess the real strength of results.

## Author Contributions

GC and EM developed the project of the study, performed the analysis, and discussed preclinical and clinical data. LC, CrM, and DQ collected and discussed clinical data. VA, CaM, and FC collected and discussed data on pharmacokinetics and pharmacodynamics.

## Conflict of Interest Statement

The authors declare that the research was conducted in the absence of any commercial or financial relationships that could be construed as a potential conflict of interest.
